# Supplementary material for: Characterization of the In Vitro and In Vivo Efficacy of Baloxavir Marboxil against H5 Highly Pathogenic Avian Influenza Virus Infection
Source: Viruses. 2022 Jan 8;14(1):111. doi: 10.3390/v14010111 (PMC8777714; doi:10.3390/v14010111)
Supplement: Supplementary file 1 [file viruses-14-00111-s001.zip › viruses-1551789-supplementary.pdf]

Supplementary data

Table S1 Details of animal experiments

Experiment 1

Examination of the survival effect in mice infected with H5N1 virus (in Figure 1 and Figure S1)

Administration	Number of mice
Vehicle (0.5 w/v% methylcellulose), twice daily for 5 days	10
OSP, 5 mg/kg/dose, twice daily for 5 days	10
OSP, 50 mg/kg/dose, twice daily for 5 days	10
BXM, 0.5 mg/kg/dose, twice daily for 1 day	10
BXM, 5 mg/kg/dose, twice daily for 1 day	10
BXM, 50 mg/kg/dose, twice daily for 1 day	10
BXM, 0.5 mg/kg/dose, twice daily for 5 days	10
BXM, 5 mg/kg/dose, twice daily for 5 days	10
BXM, 50 mg/kg/dose, twice daily for 5 days	10

Examination of inhibitory effects on viral titers and cytokines in the lungs of mice infected with H5N1 virus (in Figure 2a and 3a)

Administration	Number of mice		
	Sampling point (dpi)		
	1	3	5
Vehicle (0.5 w/v% methylcellulose), twice daily for 5 days	5	5	5
OSP, 5 mg/kg/dose, twice daily for 5 days	5	5	5
OSP, 50 mg/kg/dose, twice daily for 5 days	5	5	5
BXM, 0.5 mg/kg/dose, twice daily for 1 day	5	5	5
BXM, 5 mg/kg/dose, twice daily for 1 day	5	5	5
BXM, 50 mg/kg/dose, twice daily for 1 day	5	5	5
BXM, 0.5 mg/kg/dose, twice daily for 5 days	-	5	5
BXM, 5 mg/kg/dose, twice daily for 5 days	-	5	5
BXM, 50 mg/kg/dose, twice daily for 5 days	-	4#	5

One of 5 mice was died due to an experimental error.

Examination of inhibitory effects on viral titers in the brains and kidneys of mice infected with H5N1 virus at 6 dpi (in Figure 2b and c)

Administration	Number of mice
Vehicle (0.5 w/v% methylcellulose), twice daily for 5 days	5
OSP, 5 mg/kg/dose, twice daily for 5 days	5
OSP, 50 mg/kg/dose, twice daily for 5 days	5
BXM, 0.5 mg/kg/dose, twice daily for 1 day	5
BXM, 5 mg/kg/dose, twice daily for 1 day	5
BXM, 50 mg/kg/dose, twice daily for 1 day	5
BXM, 0.5 mg/kg/dose, twice daily for 5 days	5
BXM, 5 mg/kg/dose, twice daily for 5 days	5
BXM, 50 mg/kg/dose, twice daily for 5 days	5

Examination of prevention of inflammation in the lungs of mice infected with H5N1 virus (in Figure 3b and Figure S2)

Administration	Number of mice
Vehicle (0.5 w/v% methylcellulose), twice daily for 5 days	3
OSP, 5 mg/kg/dose, twice daily for 5 days	3
OSP, 50 mg/kg/dose, twice daily for 5 days	3
BXM, 0.5 mg/kg/dose, twice daily for 1 day	3
BXM, 5 mg/kg/dose, twice daily for 1 day	3
BXM, 50 mg/kg/dose, twice daily for 1 day	3
BXM, 0.5 mg/kg/dose, twice daily for 5 days	3
BXM, 5 mg/kg/dose, twice daily for 5 days	3
BXM, 50 mg/kg/dose, twice daily for 5 days	3
Mock	3

Examination of prevention of lung indices in mice infected with H5N1 virus (in Figure S3)

Administration	Number of mice
Vehicle (0.5 w/v% methylcellulose), twice daily for 5 days	5
OSP, 5 mg/kg/dose, twice daily for 5 days	5
OSP, 50 mg/kg/dose, twice daily for 5 days	5
BXM, 0.5 mg/kg/dose, twice daily for 1 day	5
BXM, 5 mg/kg/dose, twice daily for 1 day	5
BXM, 50 mg/kg/dose, twice daily for 1 day	5
BXM, 0.5 mg/kg/dose, twice daily for 5 days	5
BXM, 5 mg/kg/dose, twice daily for 5 days	5
BXM, 50 mg/kg/dose, twice daily for 5 days	5
Mock	5

Experiment 2

Examination of the survival effect in mice infected with H5N1 virus (in Figure 5)

Administration	Number of mice
Vehicle (0.5 w/v% methylcellulose), twice daily for 5 days	5
OSP, 10 mg/kg/dose, twice daily for 5 days	5
BXM, 5 mg/kg/dose, twice daily for 5 days	5
BXM, 50 mg/kg/dose, twice daily for 5 days	5
BXM 5 mg/kg/dose + OSP 10 mg/kg/dose, twice daily for 5 days	5

Examination of inhibitory effects on viral titers in the lungs, brains or kidneys of mice infected with H5N1 virus (in Figure 6)

Administration	Number of mice			
	Sampling point (dpi)			
	1	3	5	7
Vehicle (0.5 w/v% methylcellulose), twice daily for 5 days	5	5	5	3#
OSP, 10 mg/kg/dose, twice daily for 5 days	5	5	5	3#
BXM, 5 mg/kg/dose, twice daily for 5 days	5	5	5	5
BXM, 50 mg/kg/dose, twice daily for 5 days	5	5	5	5
BXM 5 mg/kg/dose + OSP 10 mg/kg/dose, twice daily for 5 days	5	5	5	5

Two of 5 mice in each group were died by 7 dpi.

Table S2. Quantification of viral RNA and titers from lung homogenates of BXM-treated mice

Lung ID	Administration	Sampling point (dpi)	Viral RNA (copy/reaction)	Viral titers (Log_{10} $TCID_{50}$/mL)
16	BXM, 0.5 mg/kg/dose, twice daily for 1 day	1	3.11E+03	2.67
17	BXM, 0.5 mg/kg/dose, twice daily for 1 day	1	4.84E+03	2.23
18	BXM, 0.5 mg/kg/dose, twice daily for 1 day	1	1.40E+04	3.33
19	BXM, 0.5 mg/kg/dose, twice daily for 1 day	1	3.51E+03	2.67
20	BXM, 0.5 mg/kg/dose, twice daily for 1 day	1	BLQ	1.50*
21	BXM, 5 mg/kg/dose, twice daily for 1 day	1	BLQ	1.50*
22	BXM, 5 mg/kg/dose, twice daily for 1 day	1	BLQ	1.50*
23	BXM, 5 mg/kg/dose, twice daily for 1 day	1	BLQ	1.50*
24	BXM, 5 mg/kg/dose, twice daily for 1 day	1	BLQ	1.50*
25	BXM, 5 mg/kg/dose, twice daily for 1 day	1	BLQ	1.50*
26	BXM, 50 mg/kg/dose, twice daily for 1 day	1	BLQ	1.50*
27	BXM, 50 mg/kg/dose, twice daily for 1 day	1	BLQ	1.50*
28	BXM, 50 mg/kg/dose, twice daily for 1 day	1	BLQ	1.50*
29	BXM, 50 mg/kg/dose, twice daily for 1 day	1	BLQ	1.50*
30	BXM, 50 mg/kg/dose, twice daily for 1 day	1	BLQ	1.50*
46	BXM, 0.5 mg/kg/dose, twice daily for 5 days	3	1.45E+05	3.67
47	BXM, 0.5 mg/kg/dose, twice daily for 5 days	3	4.94E+05	3.50
48	BXM, 0.5 mg/kg/dose, twice daily for 5 days	3	2.58E+04	3.23
49	BXM, 0.5 mg/kg/dose, twice daily for 5 days	3	2.25E+04	3.50
50	BXM, 0.5 mg/kg/dose, twice daily for 5 days	3	9.26E+03	2.67
51	BXM, 5 mg/kg/dose, twice daily for 5 days	3	BLQ	1.67
52	BXM, 5 mg/kg/dose, twice daily for 5 days	3	BLQ	1.50*
53	BXM, 5 mg/kg/dose, twice daily for 5 days	3	BLQ	1.50*
54	BXM, 5 mg/kg/dose, twice daily for 5 days	3	BLQ	1.50*
55	BXM, 5 mg/kg/dose, twice daily for 5 days	3	BLQ	1.50*
57	BXM, 50 mg/kg/dose, twice daily for 5 days	3	BLQ	1.50*
58	BXM, 50 mg/kg/dose, twice daily for 5 days	3	BLQ	1.50*
59	BXM, 50 mg/kg/dose, twice daily for 5 days	3	BLQ	1.50*
60	BXM, 50 mg/kg/dose, twice daily for 5 days	3	BLQ	1.50*
61	BXM, 0.5 mg/kg/dose, twice daily for 1 day	3	BLQ	1.67
62	BXM, 0.5 mg/kg/dose, twice daily for 1 day	3	4.20E+05	4.00
63	BXM, 0.5 mg/kg/dose, twice daily for 1 day	3	1.33E+06	4.50
64	BXM, 0.5 mg/kg/dose, twice daily for 1 day	3	1.62E+05	4.33
65	BXM, 0.5 mg/kg/dose, twice daily for 1 day	3	1.16E+06	4.67

66	BXM, 0.5 mg/kg/dose, twice daily for 1 day	3	BLQ	1.50*
67	BXM, 5 mg/kg/dose, twice daily for 1 day	3	2.65E+04	4.00
68	BXM, 5 mg/kg/dose, twice daily for 1 day	3	BLQ	2.77
69	BXM, 5 mg/kg/dose, twice daily for 1 day	3	BLQ	1.50*
70	BXM, 5 mg/kg/dose, twice daily for 1 day	3	BLQ	1.50*
71	BXM, 5 mg/kg/dose, twice daily for 1 day	3	BLQ	1.50*
72	BXM, 50 mg/kg/dose, twice daily for 1 day	3	BLQ	1.50*
73	BXM, 50 mg/kg/dose, twice daily for 1 day	3	BLQ	1.50*
74	BXM, 50 mg/kg/dose, twice daily for 1 day	3	BLQ	1.50*
75	BXM, 50 mg/kg/dose, twice daily for 1 day	3	BLQ	1.50*
91	BXM, 0.5 mg/kg/dose, twice daily for 5 days	5	2.18E+04	3.67
92	BXM, 0.5 mg/kg/dose, twice daily for 5 days	5	1.62E+05	3.33
93	BXM, 0.5 mg/kg/dose, twice daily for 5 days	5	8.22E+05	3.00
94	BXM, 0.5 mg/kg/dose, twice daily for 5 days	5	8.84E+04	1.50*
95	BXM, 0.5 mg/kg/dose, twice daily for 5 days	5	2.56E+05	3.50
96	BXM, 5 mg/kg/dose, twice daily for 5 days	5	BLQ	1.50*
97	BXM, 5 mg/kg/dose, twice daily for 5 days	5	BLQ	1.50*
98	BXM, 5 mg/kg/dose, twice daily for 5 days	5	BLQ	1.50*
99	BXM, 5 mg/kg/dose, twice daily for 5 days	5	BLQ	1.50*
100	BXM, 5 mg/kg/dose, twice daily for 5 days	5	BLQ	1.50*
101	BXM, 50 mg/kg/dose, twice daily for 5 days	5	BLQ	1.50*
102	BXM, 50 mg/kg/dose, twice daily for 5 days	5	BLQ	1.50*
103	BXM, 50 mg/kg/dose, twice daily for 5 days	5	BLQ	1.50*
104	BXM, 50 mg/kg/dose, twice daily for 5 days	5	BLQ	1.50*
105	BXM, 50 mg/kg/dose, twice daily for 5 days	5	BLQ	1.50*
106	BXM, 0.5 mg/kg/dose, twice daily for 1 day	5	2.29E+06	4.00
107	BXM, 0.5 mg/kg/dose, twice daily for 1 day	5	4.68E+04	2.50
108	BXM, 0.5 mg/kg/dose, twice daily for 1 day	5	1.00E+06	5.17
109	BXM, 0.5 mg/kg/dose, twice daily for 1 day	5	2.68E+06	6.50
110	BXM, 0.5 mg/kg/dose, twice daily for 1 day	5	2.96E+06	4.67
111	BXM, 0.5 mg/kg/dose, twice daily for 1 day	5	BLQ	2.50
112	BXM, 5 mg/kg/dose, twice daily for 1 day	5	BLQ	1.50*
113	BXM, 5 mg/kg/dose, twice daily for 1 day	5	BLQ	1.50*
114	BXM, 5 mg/kg/dose, twice daily for 1 day	5	BLQ	1.50*
115	BXM, 5 mg/kg/dose, twice daily for 1 day	5	BLQ	1.50*
116	BXM, 5 mg/kg/dose, twice daily for 1 day	5	BLQ	1.50*
117	BXM, 50 mg/kg/dose, twice daily for 1 day	5	BLQ	1.50*
118	BXM, 50 mg/kg/dose, twice daily for 1 day	5	BLQ	1.50*

119	BXM, 50 mg/kg/dose, twice daily for 1 day	5	BLQ	1.50*
120	BXM, 50 mg/kg/dose, twice daily for 1 day	5	BLQ	1.50*

BLQ = below the lower limit of quantification, * the lower limit of quantification of the viral titer

Table S3. Summary of variants of the PA region in viral RNAs derived from lung homogenates

Lung ID	Administration	Sampling point (dpi)	Amino acid			
			Position	Reference	Call	Minor peak*
18	BXM, 0.5 mg/kg/dose, twice daily for 1 day	1	76	F (TTT)	F (TTT)	F (TTC)
62	BXM, 0.5 mg/kg/dose, twice daily for 1 day	3	378	K (AAA)	K (AAR**)	K (AAG)
91	BXM, 0.5 mg/kg/dose, twice daily for 5 days	5	127	I (ATT)	V (GTT)	I (ATT)
94	BXM, 0.5 mg/kg/dose, twice daily for 5 days	5	574	K (AAG)	K (AAA)	K (AAG)

* Minor peaks are judged when 25% or more of the first peak is detected., ** mixed base of A and G

Table S4. PA amino acid position at 127

Influenza virus strains	Accession number*	Amino acid residue at 127
A/Hong Kong/483/1997 (H5N1)	AF084269	I
A/ruddy turnstone/Delaware/103/2007 (H5N1)	EPI296727	V
A/muscovy duck/Vietnam/OIE-559/2011 (H5N1)	EPI321743	V
A/whooper swan/Mongolia/2/2006 (H5N1)	AB263751	V
A/black swan/Akita/1/2016 (H5N6)	LC198527	V
A/northern pintail/Hokkaido/M13/2020 (H5N8)	MW228064	I
A/whooper swan/Fukushima/0701B002/2021 (H5N8)	MZ235325	I
A/whooper swan/Miyagi/0402B001/2021 (H5N8)	MZ235317	I

* Each sequence obtained from the National Center for Biotechnology Information (NCBI) and Global Initiative on Sharing All Influenza Data (GISAID) on December 26, 2021.

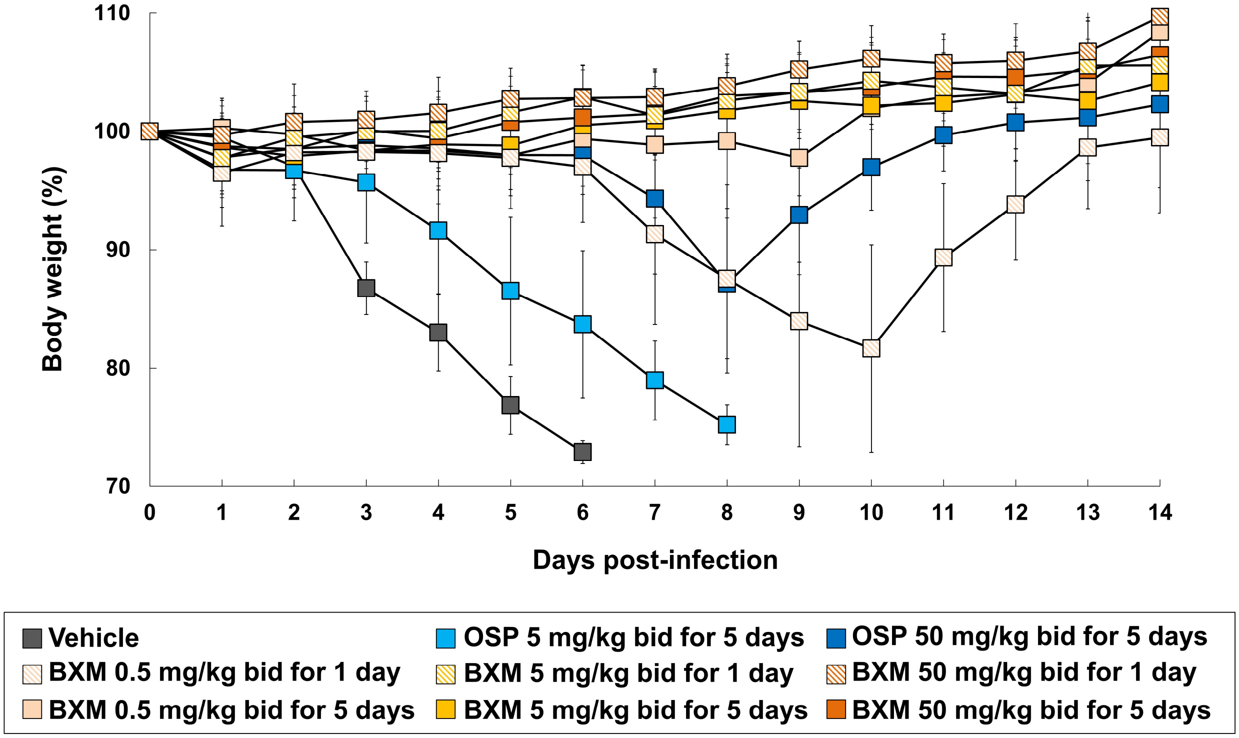

Figure S1. Therapeutic effects of BXM on body weight loss in a lethal infection mouse model with H5N1 HPAIV. Body weight changes were monitored through a 14-day period after infection. Details of mouse experiments are shown in the legend of Figure 1.

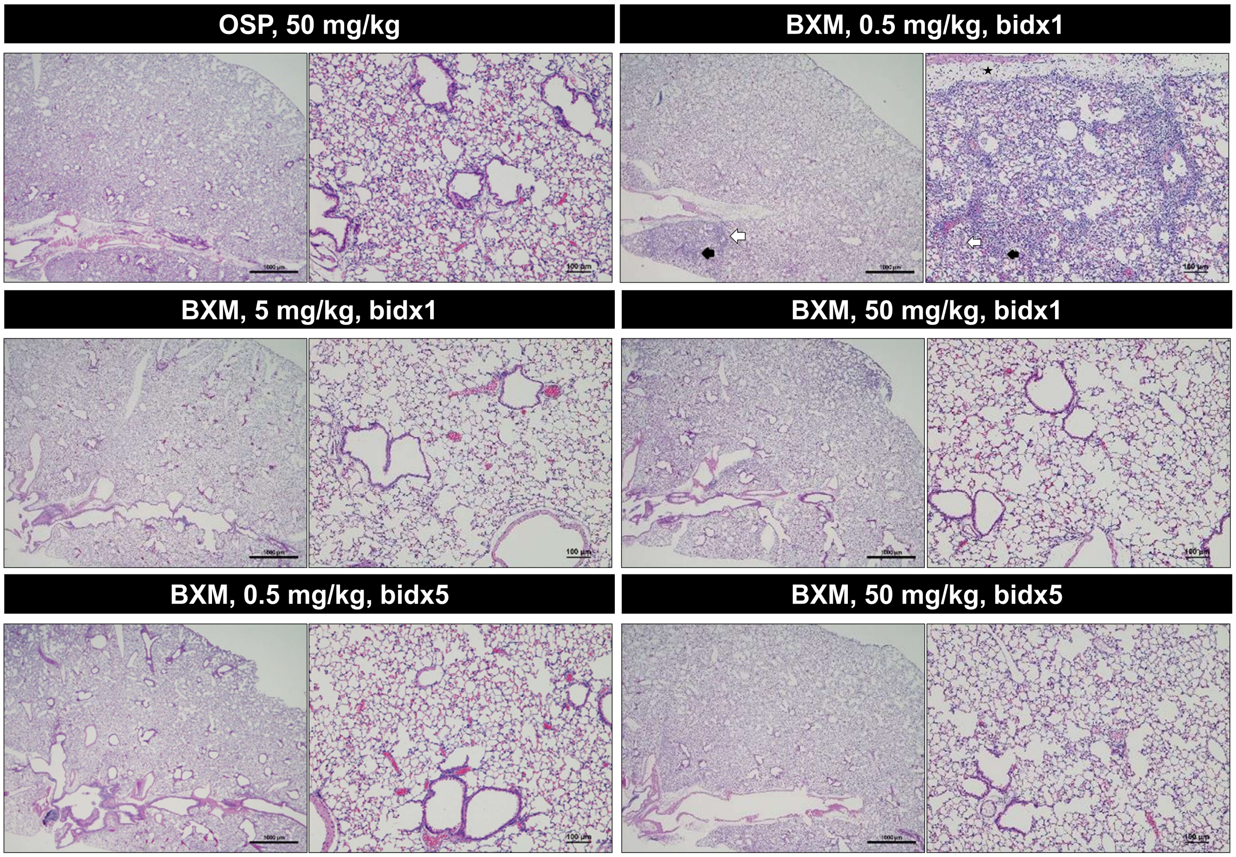

Figure S2. Histopathological findings from mice lungs following BXM treatment. The details of mouse experiments are shown in the legend of Figure 3B. Left panels of each dosing showed wide fields (2× magnification of objective), whereas right panels of each dosing showed narrow fields (20× magnification of objective). Black arrows indicate thickening and inflammatory cell infiltration of alveolar walls. White arrows indicate inflammatory cell infiltration within the alveoli. Black stars indicate edema.

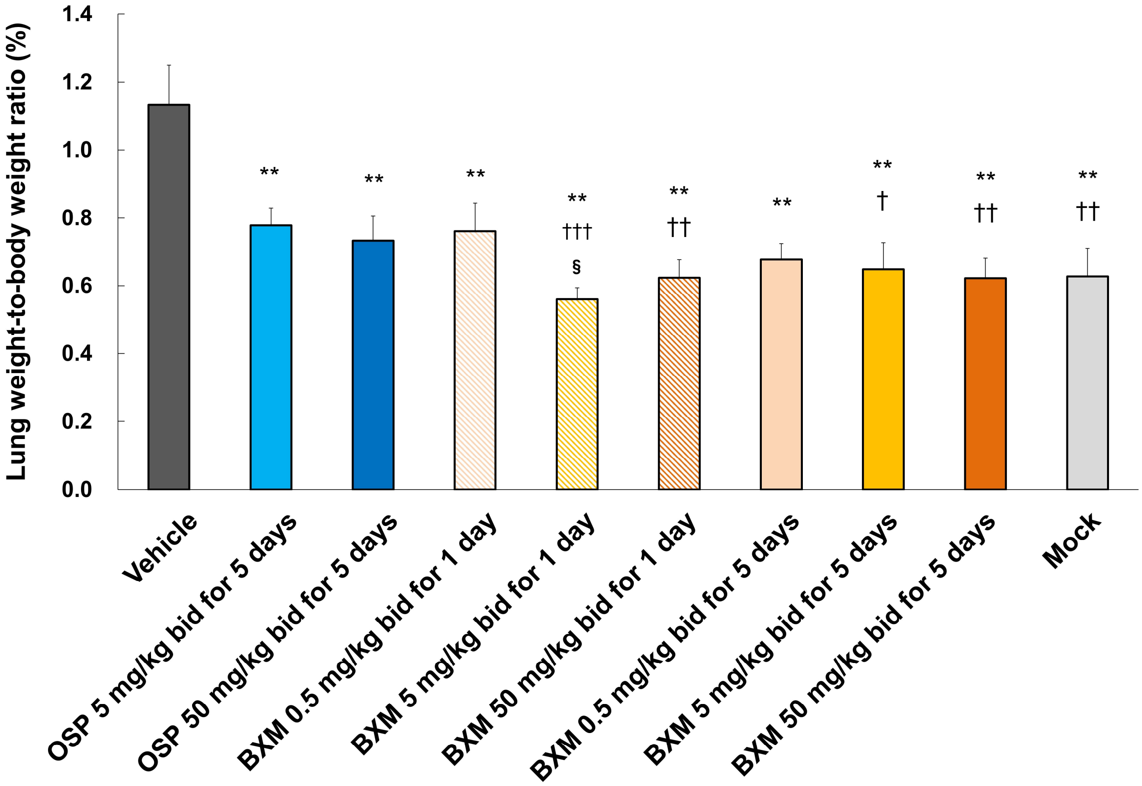

Figure S3. The prevention of lung indices in mice following BXM treatment. The body weights of mice were measured, after which whole lungs collected at 5 dpi were weighed. The lung wet weight-to-body weight ratio in each treatment group was then calculated as previously reported [44]. Dunnett's multiple-comparison method was employed for comparing the wet weight-to-body weight ratios of lungs between each group (** $p < 0.001$ compared to the vehicle. † $p < 0.05$, †† $p < 0.01$, ††† $p < 0.001$ compared to OSP at 5 mg/kg twice daily. § $p < 0.05$ compared to OSP at 50 mg/kg twice daily).
